# Induced pluripotency in the context of stem cell expansion bioprocess development, optimization, and manufacturing: a roadmap to the clinic

**DOI:** 10.1038/s41536-021-00183-7

**Published:** 2021-11-01

**Authors:** James Colter, Kartikeya Murari, Jeff Biernaskie, Michael Scott Kallos

**Affiliations:** 1 Pharmaceutical Production Research Facility, University of Calgary, 2500 University Drive NW, Calgary, AB T2N 1N4 Canada; 2 Integrated Circuits and Optical Imaging Laboratory, University of Calgary, 2500 University Drive NW, Calgary, AB T2N 1N4 Canada; 3 Biomedical Engineering Graduate Program, University of Calgary, 2500 University Drive NW, Calgary, AB T2N 1N4 Canada; 4 Department of Electrical and Software Engineering, Schulich School of Engineering, University of Calgary, 2500 University Drive NW, Calgary, AB T2N 1N4 Canada; 5 Department of Comparative Biology and Experimental Medicine, Faculty of Veterinary Medicine, University of Calgary, 2500 University Drive NW, Calgary, AB T2N 1N4 Canada; 6 Department of Surgery,Cumming School of Medicine, University of Calgary, Calgar, AB Canada; 7 Hotchkiss Brain Institute, University of Calgary, Calgary, AB Canada; 8 Alberta Children’s Hospital Research Institute, University of Calgary, Calgary, AB Canada; 9 Department of Chemical and Petroleum Engineering, Schulich School of Engineering, University of Calgary, 2500 University Drive NW, Calgary, AB T2N 1N4 Canada

**Keywords:** Induced pluripotent stem cells, Stem-cell biotechnology

## Abstract

The translation of laboratory-scale bioprocess protocols and technologies to industrial scales and the application of human induced pluripotent stem cell (hiPSC) derivatives in clinical trials globally presents optimism for the future of stem-cell products to impact healthcare. However, while many promising therapeutic approaches are being tested in pre-clinical studies, hiPSC-derived products currently account for a small fraction of active clinical trials. The complexity and volatility of hiPSCs present several bioprocessing challenges, where the goal is to generate a sufficiently large, high-quality, homogeneous population for downstream differentiation—the derivatives of which must retain functional efficacy and meet regulatory safety criteria in application. It is argued herein that one of the major challenges currently faced in improving the robustness of routine stem-cell biomanufacturing is in utilizing continuous, meaningful assessments of molecular and cellular characteristics from process to application. This includes integrating process data with biological characteristic and functional assessment data to model the interplay between variables in the search for global optimization strategies. Coupling complete datasets with relevant computational methods will contribute significantly to model development and automation in achieving process robustness. This overarching approach is thus crucially important in realizing the potential of hiPSC biomanufacturing for transformation of regenerative medicine and the healthcare industry.

## Introduction

Modern bioprocessing technology and protocols have developed to the point of producing functional, clinically relevant numbers of pluripotent stem cells for use as cell and tissue source material^1–3^. However, optimization of manufacturing protocols is resource-intensive and costly, with characterization of phenotype being laborious and discontinuous. Coupled with in-process heterogeneity and the evolving regulatory framework surrounding assessment of stem-cell-derived therapeutics, it is difficult to develop scalable, robust processes that are strictly standardized and economically viable^4,5^. Clinical outcomes are dependent on biological function of the product, with quality hindered by obstacles such as a lack of reproducibility and robustness for scale-up and scale-out^6^. Utilization of human induced pluripotent stem cells (hiPSCs) and their derivatives for drug discovery, cell therapy, and disease modeling present a potentially invaluable resource, but existing limitations to their use include conceptual biases, technological limitations, process heterogeneity, rudimentary control over cell fate, and substantial cost^7,8^.

Addressing these limitations and obstacles in the development of stem-cell-based therapeutics includes the incorporation of technologies and methods capable of continuous monitoring and assessment of phenotype throughout the bioprocess, in conjunction with process control, standardization, and automation of protocols as they are developed. These advancements will require the integration of process systems, novel analytical technologies, and computational methods throughout development and optimization. This paper aims to explore the challenges and potential strategies to overcome obstacles faced in controlling pluripotent and differentiated phenotype in the context of functionality, efficacy, and safety throughout hiPSC biomanufacturing.

## Highlighting challenges in the scalable and robust production of human iPSCs

Human pluripotent stem cells are promising candidates for therapeutic applications given their proliferative potential and capacity to differentiate into any cell type within the primary germ layers of an adult organism^9^. Naturally occurring populations of these cells are found within an early-stage embryonic blastocyst and can be isolated and expanded in vitro to generate a population of self-renewing human embryonic stem cells (ESCs)^10^. However, harvesting the cells requires destruction of the developing embryo. Utilization of hiPSCs as an alternative cell source effectively overcomes the legal, ethical, and moral barriers associated with the use of embryonic or epiblast stem cells^11^. However, conventional methods for the induction and maintenance of pluripotent state introduce a myriad of additional biological obstacles to effective application in stem-cell-derived therapeutics. These include an increased risk of mutation, retention of somatic epigenetic memory, potential for immunogenicity, and altered functional characteristics of differentiated phenotypes^12–15^.

Stem-cell research for drug development, disease modeling, and potential therapeutic applications has gained great momentum in recent years^16–18^. Progress has been made towards harnessing their curative potential and significant investment has been placed into assessment of therapeutic applications, with varying degrees of success^19–21^. The lack of success leading to suspension of many iPSC clinical trials specifically highlights a need to further elucidate the interplay between variables over the course of the bioprocess and their influence on stem-cell-derived product identity and function. Further, a lack of process robustness in conventional systems and protocols result in a high degree of in-process heterogeneity observed within batch populations and between-culture batches^22^. This heterogeneity reflects the high phenotypic plasticity of pluripotent stem cells, and the non-uniform spatiotemporal conditions observed across bioprocess systems^23^. These results are a strong reminder that process development strategies must be designed to strictly orchestrate both pluripotent and downstream phenotypes, with interventions made to minimize stochasticity and spatial gradient-induction within the process.

## Biological complexities governing iPSC phenotype and downstream functionality

Transient behavior of iPSC populations cultured in vitro are dependent on genetic variants present in the derived source cell line and the culture environment in which they are sustained. Many cultured iPSC lines present copy-number variations, with considerable and non-uniform genetic load of single-nucleotide variants among clones within a particular population^24,25^. Several identified variants affect genes implicated in cancer, and clonal dominance observed in vitro suggest selection of clones with some phenotypic advantage that while desirable for their enhanced growth characteristics may present safety risks at the clinical stage^26,27^. Coupled with chromosomal instability often observed over extended in vitro culture, these considerations highlight the need for continuity of measurement to monitor and adapt both genetic and epigenetic characteristics of the cell population.

Evidence of immune response has been observed in autologous transplantations of iPSCs in mice and raises concerns for therapeutic potential in humans^28–30^. Many such studies have focused on elucidating the effect of reprogramming strategies on genetic and epigenetic defects in the induced pluripotent cell population, and their contributions to immunogenicity in therapeutic derivatives. However, given the potential for genetic and epigenetic abnormalities to arise in culture, there is only a paucity of studies that directly assess the contribution of in vitro conditions and bioprocess variables during iPSC expansion and subsequent differentiation on immunogenic potential^31–33^.

Epigenetic mechanisms play a pivotal role in the regulation of gene expression. These mechanisms are diverse and include behaviors such as histone protein modifications and DNA methylation^34^. Epigenetic memory is defined by inherited cellular behavior as a result of prior stimulus. These alterations result in reinforcement and suppression activity in gene regulatory networks, a “learned” identity in the cell and its progeny over successive generations^35,36^. This phenomenon is particularly well-noted in iPSC populations, given their propensity for epigenetic memory as a result of the somatic source cell identity, age, metabolic signature, and reprogramming strategy used for induction of pluripotency^12,37^. Indeed, somatic memory has been shown to result in preferential bias during differentiation towards certain lineages that affects phenotype and functionality in iPSC populations and their derivatives^38^. Further, these abnormal phenotypes raise questions of cellular stability, longevity, and tumorigenic risk^39^. More work is needed to understand the long-term epigenetic landscape of iPSC populations from reprogramming all the way through to their use as constituents within a functional therapeutic, and how these factors contribute to their safety and efficacy.

**Fig. 1:**
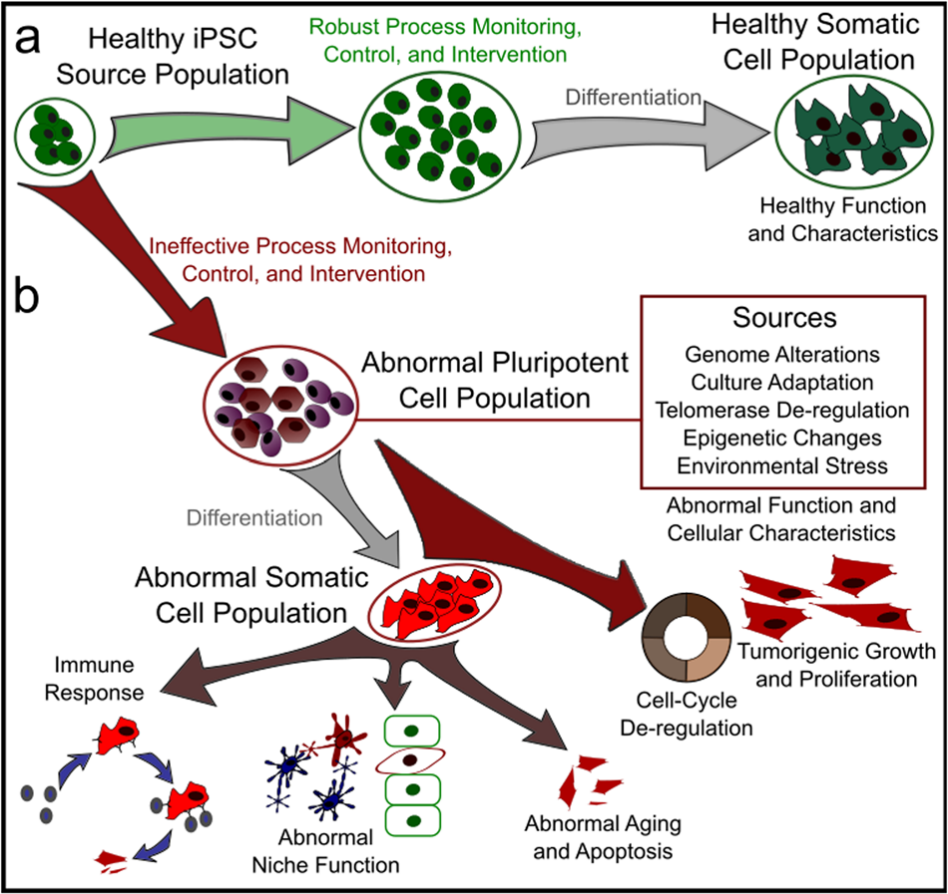
Considerations for clinical stem-cell manufacturing. **a** Ideal stem-cell population dynamics in the presence of adequate process control and intervention strategies. Maintenance of high-quality phenotype is critical in ensuring safety and efficacy in derived products. **b** Erosion of phenotype in the presence of inadequate process control and intervention, the result of which may take the form of genetic defects, elicitation of immune response by the host, abnormal function, cell cycle de-regulation, and tumorigenesis.

Functional characteristics have been reported to vary among groups studying iPSC derivatives^40,41^. Combining the implications discussed so far in this paper, it is no surprise that the biological factors responsible for variability in the reprogrammed iPSC population extend to their progeny throughout expansion and subsequent differentiation. Furthermore, biological sex differences add additional considerations—X-inactivation in female cells results in differing regulatory dynamics from XY-active male cell lines. These differences are known to lead to diverse mechanisms in cell state de-regulation and cancer progression^42–44^. Given the disparity in available data on biological sex, it is critical that researchers recognize these differences and make every effort to ensure equality in process development. While relatively few studies have focused specifically on the downstream implications of the factors discussed, it is pertinent that scientists and engineers make a concerted effort to include this information in process development and optimization, using generated data to better understand and control these overlapping cause-and-effect interactions [Fig. 1].

## Cellular interaction networks governing the pluripotent state

**Fig. 2:**
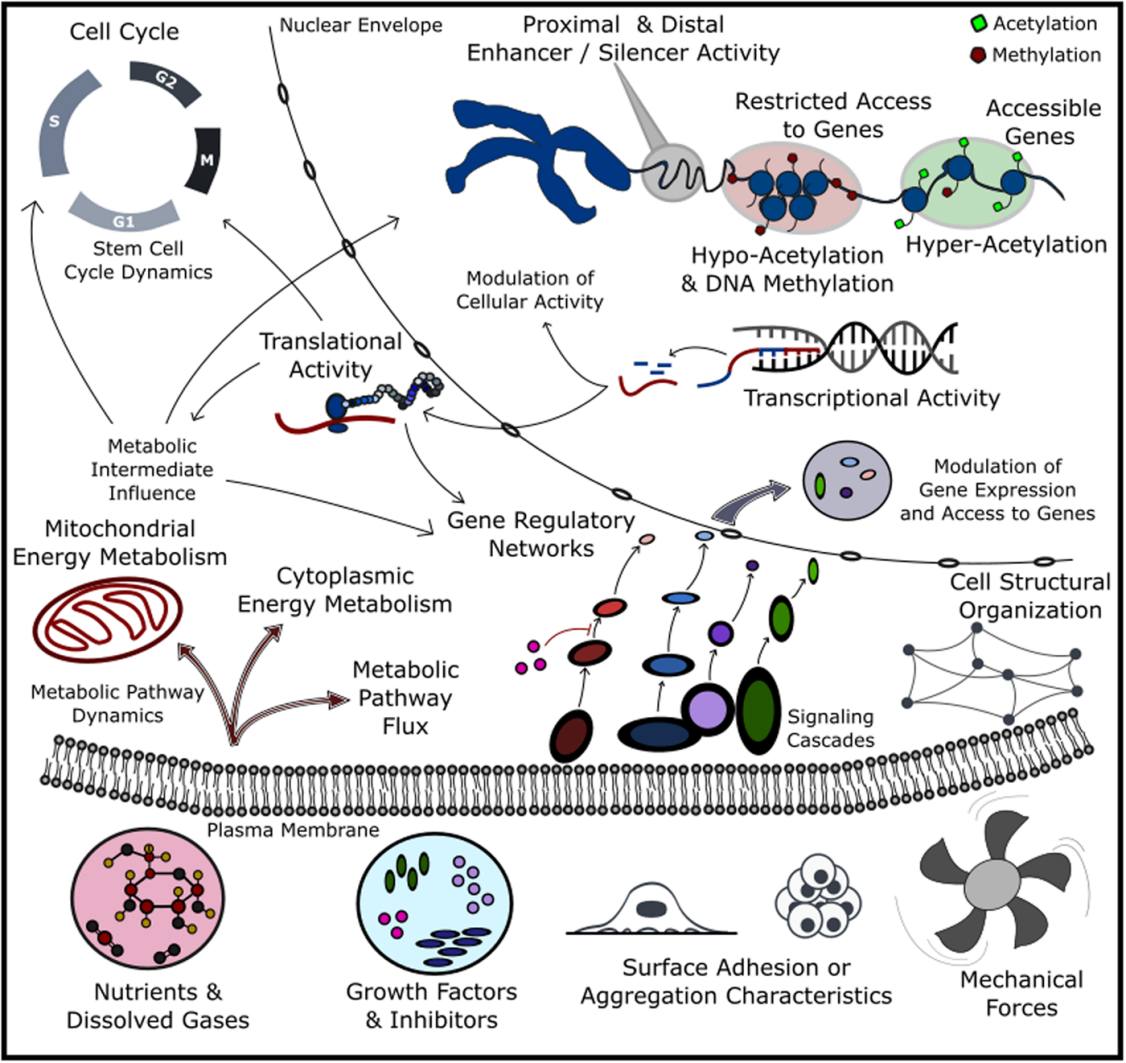
Process interaction networks at a glance. Visualization of the interaction network at a high level of abstraction. Some of the critical cellular machinery dictating cell health and phenotype are shown. This machinery interacts with one another and influences from the bioprocess to continuously maintain and influence overall quality in the process population.

The challenges discussed so far reflect the complex cellular mechanisms and network interactions that govern phenotype. The origins of these mechanisms and their implications for stem-cell-derived products must be understood to facilitate effective design and optimization of bioprocesses. Cellular phenotype is a consequence of the inherited features in the population, combined with cellular regulation and environmental niche responses. This section will describe these interactions and the results of recent work to understand and control phenotype with regard to their respective mechanisms. Arguably, the interaction network arising from complex multi-system behavior within the cell results in a co-regulatory dynamic that is critical to the maintenance of pluripotent phenotype [Fig. 2].

DNA methylation is known to play an important role in restricting genetic access by transcriptional machinery^45^. In naïve state pluripotency, hypomethylation of the global genome is observed^46^. As the cell transitions towards a primed state, hypermethylation of the genome occurs, an orchestrated restriction of access to genes by epigenetic machinery. IPSCs exhibit unique methylation patterns as a result of reprogramming, with outcomes dependent on source cell characteristics and the strategy used^47^. This somatic memory is a form of genomic imprinting and may result in aberrant patterns of activity not exhibited in naturally occurring pluripotent stem cells. Further, contributions to the genetic landscape from clonal inheritance through passaging have been shown to play an important role during reprogramming, expansion, and subsequent differentiation of the cell population^28,48,49^.

Pluripotent stem-cell populations exhibit unique chromatin activity that is fundamental to their plasticity^50^. In the earliest stages of pluripotency, nuclear chromatin in the naïve-state pluripotent cell maintains open euchromatin structures, supporting an enhanced ability of these cells to respond to a wide array of developmental signaling cues^51^. As these cells become primed to differentiate, interactions within gene regulatory networks result in a repressive heterochromatin state, diminishing cell potency in combination with other mechanisms as the cell transitions to a further developed lineage-committed phenotype. These distinct histone protein modifications and gene methylation patterns govern gene accessibility to the transcriptional machinery of the cell^52^. These phenomena are significant given the influence they exert over gene expression. However, they constitute a subset of mechanisms within the overall interaction network governing iPSC phenotype and are influenced by both genetic and metabolic activity within the network^53^.

Several networks have been identified as critical in sustaining the pluripotent state. It is important to highlight that these networks differ depending on the developmental state of the cell^53^. While the key pathways regulating Oct4, Sox2, and Nanog are important across pluripotent phenotypes, the naïve state is associated with gene expression activity distinct from the primed state, with differential regulation of TFCP2L1, OTX2, DUSP6, ZIC, DPPA, and Klf genes recently reported as indicators of pluripotent state^51^. Interactions between the cell and environmental niche modulate their expression, while also exerting influence over the expression of genes governing cell cycle and metabolic pathway activity^54,55^. This genetic modulation is enabled via key pathways including FGF, LIF, TGF-β, WNT, IGF, and BMP^56^. These pathways influence their target genes directly, but also cohesively and antagonistically interact with one another, resulting in complex activation and inhibition activity that guides cell phenotype in the context of pluripotency.

Metabolic intermediates also play important roles in cell state homeostasis and fate commitment^57^. Alpha-ketoglutarate is a prime example, as it inhibits histone and DNA methylation through its role in the upregulation of jumonji-c (JmjC) domain histone demethylase and ten-eleven translocation methylcytosine dioxygenase (TET1) acetyl mark deposition^58^. Similarly, acetyl Coenzyme A has been shown to contribute to the activation of histone acetyltransferase (HAT). Further, nicotine adenine dinucleotide (NAD+) activity is associated with SIRT1 upregulation^59^. These types of behaviors are intuitive in the discussion of cell phenotype, given the shifts in mitochondrial activity and dominant metabolic pathway activity observed as cell state shifts from naïve towards a primed pluripotent state, and onwards into lineage commitment^60^. This also raises questions about the extent to which the metabolic abnormalities present in iPSCs influence phenotype—a contributing factor that has yet to be fully explored^48^.

**Fig. 3:**
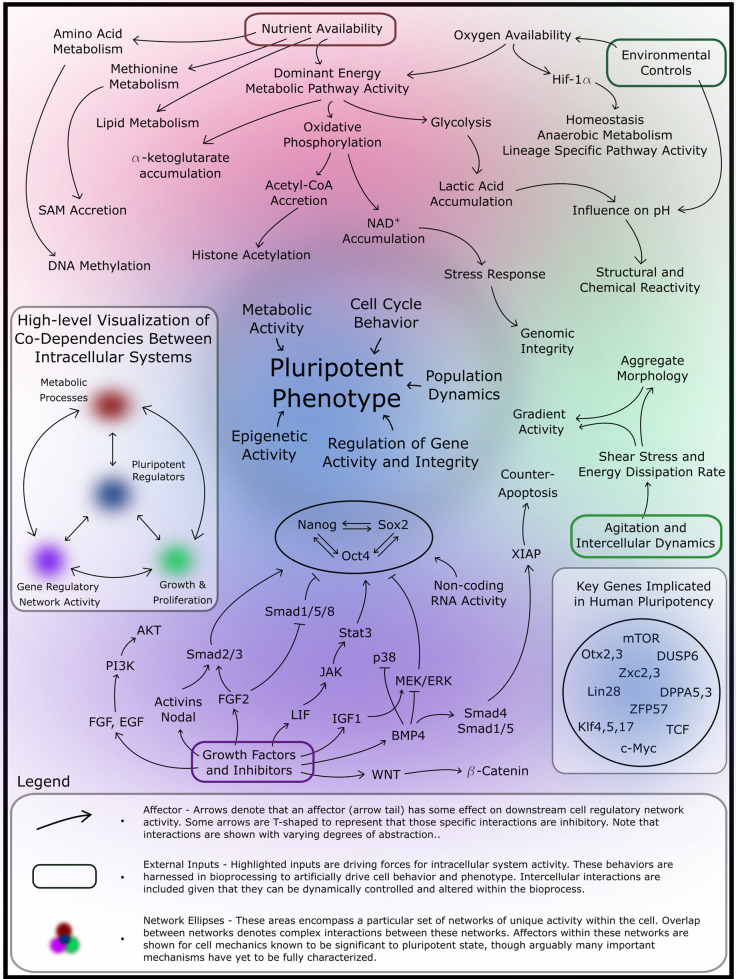
Complex interplay between internal regulatory processes and the influence of local environmental cues. Orchestration of phenotype is complex, depicted above in a conceptual interaction network. These interactions are illustrated to visualize the interplay between fundamental cellular processes. Ideal process control must be optimized to maintain strict homeostasis of the embryonic niche in pluripotent cell expansion bioprocessing by guiding cell network activity.

This evidence indicates that maintenance and transition of the cell state is highly dependent on metabolic network activity. In parallel, gene regulatory networks (GRNs) contribute to regulation of epigenetics and gene expression. Coupling metabolism with GRNs and regulation of the epigenetic structure of DNA within the cell results in a complex interaction network. These variables must be considered in the development and optimization of systems and protocols to produce safe, effective stem-cell-derived therapeutics. A number of these variables, their influences, and the implication for phenotype and cell health are visualized below [Fig. 3].

## Interplay between cell state and the bioprocess environment

Given the preceding discussion regarding the various factors influencing adoption or maintenance of cell phenotype, the obvious question becomes how to effectively control these events within the context of clinical manufacturing. Lessons in developmental biology have elucidated to some extent the mechanisms driving manifestation of the pluripotent state and its subsequent loss as a combination of cellular programs and influence by the dynamic niche environment^61^. Methodologies to reprogram, maintain, expand, and differentiate pluripotent cell populations have borrowed heavily from the mechanisms governing embryonic development. Reprogramming and differentiation strategies will not be discussed herein, although the reader is encouraged to explore several relevant reviews^62,63^.

Maintenance of the pluripotent state and successful large-scale expansion of a pluripotent population requires careful design and implementation of the process environment, with considerations made for the entirety of the cell product pipeline. The most effective systems for large-scale expansion maximize the available space for cell cultivation. Three-dimensional stirred-tank environments are a strong choice for expansion given their spatial optimization, maintenance of a well-mixed environment, and feasibility for inclusion of sensing modalities^5^. This is particularly important in maintaining dissolved gas, signaling factor, and nutrient concentration throughout the vessel.

The addition of shear stress and vorticity presents both complication and opportunity given evidence of a supporting role for mechanical forces in stimulating pathway activity, affecting growth rates, and influencing aggregate formation^64^. Influence over aggregate formation is paramount given the gradients observed throughout individual aggregates. While microcarriers present an alternative approach to minimize gradients, they reduce cultivation capacity and present additional downstream purification challenges^5,64^.

Pathway stimulation, stochastic behavior, and gradient induction are important concepts in stem-cell bioprocessing. In vivo, the niche environment undergoes dynamic spatiotemporal changes during development that result in distinct oxygen, nutrient concentrations, and growth factor composition as the cells transition out of pluripotency^61^. This is important to highlight given that their plasticity extends to in vitro culture and is reflected in the distinct results observed in varied process implementations. Media formulations have been developed to regulate primed or naïve pathways in pluripotent stem cells, with varied results and prominent population heterogeneity^65^. While great effort has been placed in derivation of optimized media composition and replacement protocols, studies on dissolved oxygen availability, energy dissipation, and nutrient balance have only recently gained momentum in the field. Environmental oxygen is particularly interesting given the evidence of interactions of HIF family proteins with the epigenetic framework of the cell, and their direct transcriptional regulation of target genes involved in metabolism and cell cycle^66^. Modulation of available oxygen plays a role in growth and dominant metabolic activity of the population, an important consideration given the overall interaction network governing the state and health of the process population^67^.

## Monitoring and characterization of pluripotent cell populations

What are the defining characteristics of a high-quality, safe, and effective pluripotent stem-cell product? Given the evidence presented, this definition will continue to evolve as researchers further elucidate mechanisms involved in the interaction network. Assessment of phenotype and function from a manufacturing perspective must balance between quantity and quality information, cost, process invasiveness, time to results, and sample representation of the population. This balancing act must satisfy safety and efficacy requirements in a clinical context and provide sufficient information to guide process development and optimization. Further, this information is necessary to assess cause-and-effect in subsequent differentiation and application.

The reader is referred to several techniques commonly used to assess the genome with varying levels of resolution^68^. Choice of technology and time points for repeated measurement should consider process dynamics, as well as the evidence for culture adaptation, mutability, and clonal dominance over the course of process progression^26,27^. While FACS analyses of surface and intracellular markers of pluripotency are commonly used, the exact targets of these analyses with respect to naive versus primed phenotypes continue to change as scientists uncover a more complete understanding of pluripotent state^51^. Further, the qualitative nature of high-throughput systems such as FACS makes it difficult to assess relative expression levels of key indicators of cell state transition within pluripotent populations.

Outside of targeted studies, dominant metabolic pathway activity has garnered significantly less attention in the bioprocessing sector. With evidence to suggest synergy between metabolism, epigenetic activity, and gene expression in stem-cell populations, a more complete focus on cell state should include integrated analysis of continuous in-process measurements and repeated offline characterization of the environment and cell population. Advancement in mass spectrometry systems and the momentum gained by online systems to assess dissolved gas and metabolic analytes has provided new opportunities to integrate these data alongside conventional characterization strategies.

It is still unclear what role metabolism and epigenetics play in the context of downstream function and phenotype, though the effect of donor variance on process heterogeneity suggests that methylation and acetylation data could go a long way in elucidating potential focal points for the research community, when combined with other characteristic and functional data. Functional assessments of human pluripotent stem cells exist, with varying degrees of rigor, dependent on the downstream application. While this paper will not present these methods in detail, it is important that interpretation of the results considers their limitations^69^.

## Bioprocess design and the therapeutic cell product

Given the infancy of stem-cell-derived product usage in therapeutic application, there remain many unknowns not only from a fundamental science perspective but also in terms of safety and efficacy for clinical use^70,71^. Maintaining a balance within the environmental niche in which the population is induced, expanded, differentiated, and maintained prior to cell product use is paramount^72^. Given the large number of cells required for a single patient (10^6^–10^12^) this balance becomes even more precarious when coupled with the necessity to expand, maintain, and influence large quantities of cells in the most resource-constrained (efficient) way possible^5^. Media formulations and shear stress application focused on maximum cell replication and expression of a small number of phenotypic markers are not sufficient to guarantee quality in the resultant cell population^73^.

These design considerations raise important questions about process specifications and the quality control metrics used throughout the entirety of the process. The choice of whether to use a surface-adherent or suspended aggregate approach inevitably impacts the cell population. The culture surface (or lack thereof) influences structural and morphological characteristics of the cell and alters gene regulatory pathways that are implicated alongside other pathways in order to balance phenotype^74–76^. This in conjunction with intracellular organization results in the gradient dynamics discussed earlier in this paper. Further, proliferation capacity and ability to sense and control the environmental niche are constrained by the process system used, affecting fluid dynamic setpoints, feeding regimen, timelines, and characterization strategies.

**Fig. 4:**
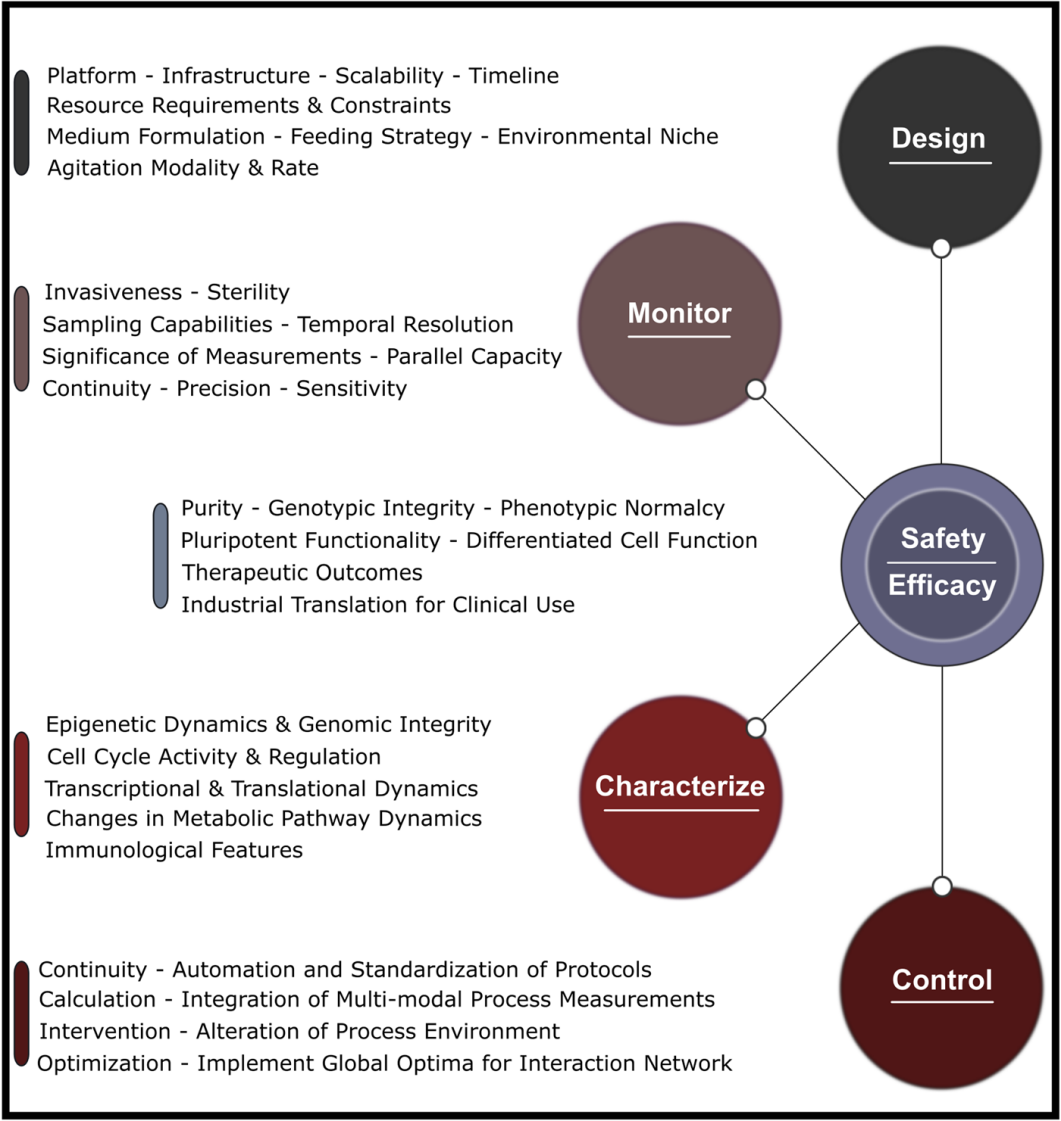
Overview of several important components and characteristics in bioprocess design, development, and optimization. Considerations must be made for the infrastructure and technology used to monitor, control, and intervene in the process. These considerations must be integrated with those made for characterization of the cell population to build a complete model for artificial manipulation of phenotype to ensure safety and efficacy downstream of expansion.

Given the spatial constraints of static systems, the strategy used to generate clinically relevant cell product numbers involves scaling out cell production by culturing replicates of a particular vessel in parallel^77^. Alternatively, dynamic suspension culture systems enable scale-up, increasing the capacity of the system within a singular vessel^78^. Both fundamental modalities include their own challenges, requiring assessment of resource constraints, bioprocess efficiency, robustness, reproducibility, capacity for monitoring, control, intervention, and tolerance of the process to donor variability and biological heterogeneity^74–76,78^. Considerations must also be made for sustainability and cost, as growing challenges for the future. A summary of these considerations is presented in Fig. 4.

Conventional bioprocess trajectories are operator and protocol dependent, with relatively basic interventions occurring within the process itself^5^. Modern process development infrastructures generate large quantities of meaningful environmental data relevant to cell population dynamics, and these data are commonly used in a closed-loop, automated configuration to maintain pH, oxygen, and perfuse the culture medium. This environmental data are coupled with rigorous characterization through multi-omics approaches to characterize population phenotype throughout the developmental stages of a process^68,73^. However, there is generally a disconnect between how offline omics and in-process data are utilized, with datasets independently scrutinized to assess a particular characteristic of the process or cell population. The two have yet to be meaningfully integrated.

Advances in machine learning have provided the means to assess large quantities of multi-dimensional datasets with the aim of developing predictive models and intervention strategies for biological systems^79–81^. There exist unique opportunities to apply concepts in machine learning and computational modeling to advance stem-cell bioprocess development and clinical manufacturing. Several works within basic research and clinical biomanufacturing spheres have been published with this mindset, though the field of iPSC biomanufacturing has yet to rigorously explore these opportunities^82–84^. Robust iPSC expansion and differentiation processes are closed-loop systems, and the incorporation of predictive models and real-time intervention strategies has the potential to greatly enhance predictability and online process control over cell health and phenotype, resulting in safer and more effective iPSC-derived cell therapeutics.

## Clinical trials and the current state of iPSC-derived therapeutics

Safety and efficacy remain key challenges to overcome in the utilization of pluripotent cell-derived therapeutics for routine use in clinical applications. This is represented in the lack of interventional studies using ESCs or iPSCs as the derivational source relative to other cell types^85^. As of August 2021, only 1.2% of documented stem-cell-derived therapeutic clinical trials in the United States alone utilized iPSCs. This number is even lower for ESCs, at 0.8%. Further, the vast majority of trials utilizing pluripotent stem cells involve in vitro disease modeling. While these studies exemplify the importance of PSCs in medicine and underline critical steps in the derivation of therapeutics for clinical application, the lack of interventional studies utilizing autologous or allogeneic PSC-derived cell therapies highlight the work that is still needed to bring them to the forefront of modern medicine. Recent work to spotlight interventional studies involving PSC-derived therapeutics show some promising progress and results in the sphere of PSC utilization^86^. A number of these trials have been further reviewed to bring clinical context to the challenges and recommendations outlined in this paper [Table 1].

**Table 1:** Bioprocess characteristics and intervention outcomes for clinical trials involving pluripotent stem-cell-derived therapeutics.

ID number	Derived cell type	Bioprocess characteristics	Manufacturing timeline	Population characterization strategies	Clinical outcomes/trial status
NCT01625559	hESC-derived retinal pigment epithelium (Allogeneic)	hESC on mouse-feeder in static 2D culture, differentiation strategy not disclosed, in-process monitoring not disclosed	Not disclosed	Morphology, cell counts, pigmentation, karyotyping (g-banding), FACS (hPSC/hRPE markers), cell function assays, short tandem repeat analysis, sterility testing, mycoplasma testing, endotoxin analysis, GMP compliance	Phase I complete, results deemed favorable^87^
NCT02923375	iPSC-mesanchymoangioblast-derived mesenchymal stem cells (allogeneic)	Cymerus platform (2D system) in serum-free, feeder-free conditions, in-process monitoring not disclosed^93^	Not disclosed	mRNA qPCR, antibody staining flow cytometry, comparative genomic hybridization, single-nucleotide polymorphism analysis, global transcriptome analysis, GMP-compliant manufacturing	Phase I complete. Short-term safety verified. Confounding factors obscured potential effectiveness^89^
NCT02057900	hESC-derived cardiovascular progenitors (allogeneic)	hESC on mouse-feeder in static 2D culture, treatment of cultures with BMP2 + SU5402 medium, downstream filtration of SSEA-1+ cells, in-process monitoring not disclosed	Not disclosed	Morphology, cell counts, marker staining, microscopic imaging, FACS (hESC/cardiac progenitor markers), HLA phenotyping, GMP compliance	Phase I complete, short-term safety verified, slightly improved symptomatic results^94^
JPRNUMIN000011929	iPSC-derived retinal pigment epithelium sheets (autologous)	2D hiPSC culture, differentiation to hiPSC-RPE, seeding onto collagen type I gel, formation of monolayer, in-process monitoring not disclosed	6 weeks to RPE sheet formation from hiPSC-RPE	Morphology, pigmentation, RT-PCR for RPE markers, assays for growth factors (ELISA), microarray genomics, MHC histocompatibility testing, DNA methylation testing, copy-number variation analysis, GMP compliance^95^	Trial halted after 2 patients. Deemed ineffective, with safety concerns^88^
NCT03482050	ESC-derived astrocytes (allogeneic)	Not disclosed	Not disclosed	Not disclosed	Trial complete, no results posted
NCT03119636	ESC-derived neural precursor cells (NPCs) (allogeneic)	EB neural differentiation strategy, not effectively disclosed for pre-clinical human ESC-derived NPCs in primates^96^	3 weeks to early neural rosette formation	Not disclosed	Unknown status

Available data from interventional clinical trials utilizing PSC-derived therapeutics present mixed results from safety and efficacy standpoints. While a recent trial by Sung et al.^87^ reported short-term safety and improved outcomes as a result of treatment with ESC-derived retinal pigment epithelium (RPEs), a similar strategy utilizing iPSC-derived RPEs resulted in trial suspension over safety concerns^88^. The most prominent difference between these two trials is the use of allogeneic ESC-derived RPEs in the Sung trial, versus autologous iPSC-derived RPEs in the Mandai trial. While confounding variables exist in the clinical application stages, a critically important detail is the presence of copy-number variations in iPSC-derived RPEs revealed during genomic analysis, resulting in trial suspension citing safety concerns in adherence with Japanese iPSC laws. Considering the similarity in their downstream use of two-dimensional static culture systems, well-established protocols, and current good manufacturing practice (CGMP), observation of genomic aberrations highlights the complexity of the iPSC pipeline from harvest, reprogramming, and expansion, through to target cell derivation. While allogeneic iPSC-derived therapeutics have recently been shown to exhibit short-term safety in the derivation of mesenchymal stem cells for host-versus-graft disease, they pose innate immune rejection risks of their own, resulting in suppression of the host immune-system following transplantation^89^. Whether allogeneic iPSCs present a more favorable cell source material remains to be seen in a clinical context.

A lack of public disclosure in the majority of clinical trials is evident, with very few trials citing publications with reference to pre-clinical work, bioprocess strategies, characterization specifics, manufacturing details, or other pipeline information. In cases where public disclosure is provided, details are scarce. Aside from trial-specific information, publication of data to publicly accessible databases is at the discretion of the trial leads and in many cases is not available^85^. This unfortunately makes an objective comparison of manufacturing design, monitoring, control, and characterization across trials near impossible to perform. A set of minimal quality criteria does exist for constitution of a clinical-grade iPSC-derived therapeutic throughout the pipeline, and while a framework exists for therapeutic generation through CGMP there are no standardized requirements for systems implemented and protocols used, leading to differing process effectiveness, robustness, and outcomes^90,91^. While most documented trials involve conventional two-dimensional static systems for both PSC and differentiation strategies, the adoption of three-dimensional, computer-controlled systems has resulted in a paradigm shift both for PSC maintenance and expansion as well as differentiation where cell types and current differentiation strategies permit. Coupled with increased capacity for process monitoring in process environments, pending trials currently in the recruitment and active stages are expected to provide far greater detail and insight into process influence on therapeutic safety and efficacy in a clinical context across systems^92^. While a lack of standardization across the field drives innovation in biomanufacturing, methods to compute the dynamics of interactions between process and target cell population will inevitably be required to assess their interplay, and disclosure of data necessary to objectively identify best practices as the field evolves.

## Conclusion and recommendations for the future

The global stem-cell research community has made leaps and bounds in the pursuit of knowledge pertaining to cellular and niche control over stem-cell phenotype. In parallel, pressure for novel medical approaches to cure disease and industrial push to implement clinical-scale engineering solutions have helped drive stem-cell science and technology over the last several decades. The number of active stem-cell-derived products in clinical trials has increased exponentially, and biopharmaceuticals companies have taken advantage of work on organoid development and disease modeling to amplify drug discovery capabilities by improving experimental capacity and scalability.

Given the interdisciplinary nature of the field and the pressure for clinical success, advancement is continuing across sectors, ranging from technological developments to increased-scale bioprocessing and clinical infrastructures. Despite these advancements, the challenges discussed in this paper have yet to be overcome in pursuit of safe and effective cell and tissue therapies to cure disease and regenerate tissue after injury. The mechanisms governing pluripotent phenotype are complex, and indicators of cell population quality extend far beyond marker presence or capacity for self-renewal and differentiation. Further, the mechanisms contributing to variance and uniqueness in pluripotent populations extend to their progeny and differentiated cell types.

Consolidating the nexus of gene expression, epigenetic activity, metabolism, cell cycle, and cell structural organization is crucial to properly influence phenotype to minimize variance and maximize cell quality. Integrating the full range of considerations and compiling more rigorous characterization and process analytics datasets across the process pipeline are necessary to enable advanced systems design. Expanding on these insights from a clinical perspective by integrating advanced medium formulations, process control strategies, online monitoring, real-time interventions, and automation alongside technological innovations will push the threshold of our capabilities to adapt iPSCs for widespread medical use in the coming decades. Coupling technology with rigorous clinical outcome measures in patients will ultimately allow the scientific community to ascertain and guide fate, function, safety, and efficacy.

## Data Availability

Any and all data are available upon request to the corresponding author.
